# Competing Mechanisms of Stress-Assisted Diffusivity and Stretch-Activated Currents in Cardiac Electromechanics

**DOI:** 10.3389/fphys.2018.01714

**Published:** 2018-12-03

**Authors:** Alessandro Loppini, Alessio Gizzi, Ricardo Ruiz-Baier, Christian Cherubini, Flavio H. Fenton, Simonetta Filippi

**Affiliations:** ^1^Unit of Nonlinear Physics and Mathematical Modeling, Department of Engineering, University Campus Bio-Medico of Rome, Rome, Italy; ^2^Mathematical Institute, University of Oxford, Oxford, United Kingdom; ^3^Laboratory of Mathematical Modelling, Institute of Personalized Medicine, Sechenov University, Moscow, Russia; ^4^ICRANet, Pescara, Italy; ^5^Georgia Institute of Technology, School of Physics, Atlanta, GA, United States

**Keywords:** cardiac electromechanics, stress-assisted diffusion, stretch-activated currents, finite elasticity, reaction-diffusion

## Abstract

We numerically investigate the role of mechanical stress in modifying the conductivity properties of cardiac tissue, and also assess the impact of these effects in the solutions generated by computational models for cardiac electromechanics. We follow the recent theoretical framework from Cherubini et al. (2017), proposed in the context of general reaction-diffusion-mechanics systems emerging from multiphysics continuum mechanics and finite elasticity. In the present study, the adapted models are compared against preliminary experimental data of pig right ventricle fluorescence optical mapping. These data contribute to the characterization of the observed inhomogeneity and anisotropy properties that result from mechanical deformation. Our novel approach simultaneously incorporates two mechanisms for mechano-electric feedback (MEF): stretch-activated currents (SAC) and stress-assisted diffusion (SAD); and we also identify their influence into the nonlinear spatiotemporal dynamics. It is found that (i) only specific combinations of the two MEF effects allow proper conduction velocity measurement; (ii) expected heterogeneities and anisotropies are obtained via the novel stress-assisted diffusion mechanisms; (iii) spiral wave meandering and drifting is highly mediated by the applied mechanical loading. We provide an analysis of the intrinsic structure of the nonlinear coupling mechanisms using computational tests conducted with finite element methods. In particular, we compare static and dynamic deformation regimes in the onset of cardiac arrhythmias and address other potential biomedical applications.

## 1. Introduction

Cardiac tissue is a complex multiscale medium constituted by highly interconnected units, cardiomyocytes, that conform a so-called *syncitium* with unique structural and functional properties (Pullan et al., 2005). Cardiomyocytes are excitable and deformable muscular cells that present themselves an additional multiscale architecture in which plasma membrane proteins and intracellular organelles all depend on the current mechanical state of the tissue (Salamhe and Dhein, 2013; Schönleitner et al., 2017). Dedicated proteic structures, such as ion channels or gap junctions, rule the passage of charged particles throughout the cell as well as between different cells and they are usually described mathematically through multiple reaction-diffusion (RD) systems (Cabo, 2014; Dhein et al., 2014; Kleber and Saffitz, 2014). All these coupled nonlinear and stochastic dynamics, emerge then to conform the coordinated contraction and pumping of the heart (Augustin et al., 2016; Land and et. al., 2016; Quarteroni et al., 2017). During the overall cycle, the mechanical deformation undoubtedly affects the electrical impulses that modulate muscle contraction, also modifying the properties of the substrate where the electrical wave propagates. These multiscale interactions have commonly been referred in the literature as the mechano-electric feedback (MEF) (Ravelli, 2003). Experimental, theoretical and clinical studies have been contributing to the systematic investigation of MEF effects, already for over a century; however, several open questions still remain (Quinn et al., 2014; Quinn and Kohl, 2016; Land et al., 2017; Sack et al., 2018). For example, and focusing on the cellular level, it is still now not completely understood what is the effective contribution of stretch-activated ion channels and which is the most appropriate way to describe them. In addition, and focusing on the organ scale, the clinical relevance of MEF in patients with heart diseases remains an open issue (Orini et al., 2017), and more specifically, how MEF mechanisms translate into ECGs (Meijborg et al., 2017) and what is the specific role of mechanics during cardiac arrhythmias (Christoph et al., 2018).

The theoretical and computational modeling of cardiac electromechanics has been used to investigate some key aspects of general excitation-contraction mechanisms. For instance, the transition from cardiac arrhythmias to chaotic behavior, including the onset, drift and breakup of spiral/scroll waves (Panfilov and Keldermann, 2005; Bini et al., 2010; Keldermann et al., 2010; Dierckx et al., 2015), pinning and unpinning phenomena due to anatomical obstacles (Cherubini et al., 2012; Hörning, 2012; Chen et al., 2014), as well as the multiscale and stochastic dynamics both at subcellular, cellular and tissue scale (Trayanova and Rice, 2011; Hurtado et al., 2016; Land et al., 2017). However, the formulation of MEF effects into mathematical models has been primarily focused on accounting for the additive superposition of an active and passive stress to stretch-activated currents (Panfilov and Keldermann, 2005). Recent contributions have advanced an energy-based framework for the comparison of active stress, stretch-activated currents and inertia effects (Cherubini et al., 2008; Ambrosi and Pezzuto, 2012; Rossi et al., 2014; Costabal et al., 2017). These works further highlight the role of mechanics into the resulting heart function at different temporal and spatial scales.

**Figure 1:**
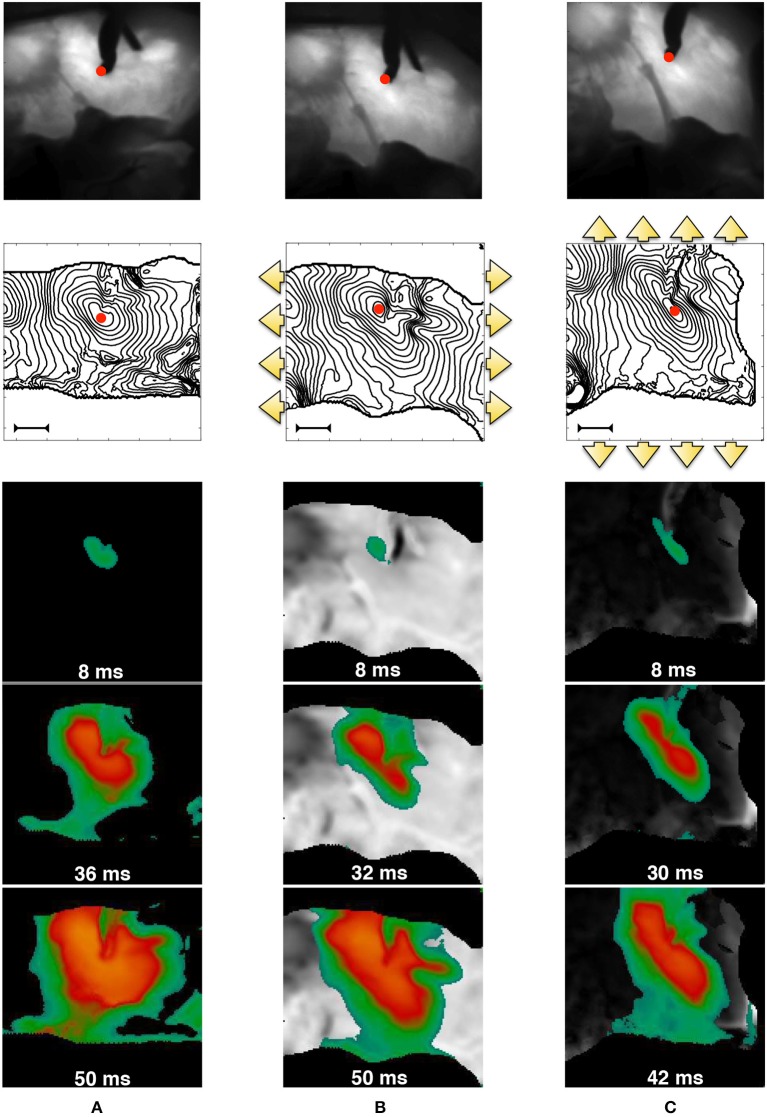
MEF observed in pig right ventricle via fluorescence optical mapping. From top to bottom, we provide: underlying tissue structure in reference **(A)** and deformed **(B,C)** states; activation isochrones each 4ms originating from the stimulation point (red spot in the field of view–the bar indicates a length of 1cm), and activation sequences. The three cases refer to no-stretch **(A)**, static horizontally **(B)**, and vertical **(C)** stretch in the directions indicated by the yellow arrows. The sequence of spatial activation uses the color code scaled to the AP level (yellow/green–high/low). Selected frames highlight the anisotropy induced by stretch. The outer black region is the noisy area not useful for the field of view.

In order to further motivate our theoretical developments, we provide an experimental representative example of the strong MEF coupling in cardiac tissue, observable on the macroscale. The data shown in Figure 1 were obtained via dedicated fluorescence optical mapping applied on a pig right ventricle (the experimental procedure has been previously described in Fenton et al., 2009; Gizzi et al., 2013; Uzelac et al., 2017). After motion suppression via blebbistatin, the perfused tissue was electrically stimulated via an external bipolar stimulator with strength twice diastolic threshold. An excitation pulse with constant pacing cycle length of 1s was delivered within the field of view (red spot in Figure 1) for several seconds (reaching a steady-state configuration) and for three different mechanical loading conditions on the same wedge: (a) free edges, (b) static uniaxial horizontal stretch, (c) static uniaxial vertical stretch with respect to a prescribed tissue orientation. The figure displays the underlying structure with clear evidence of the deformed tissue architecture, isochrones of electrical activation for a representative stimulus, and a sequence of spatial activation maps, where the colors indicate the level of activation–Action Potential (AP). Since in this proof of concept setup active contraction is inhibited by blebbistatin, these experiments clearly indicate that an additional degree of heterogeneity and anisotropy appears in the tissue and affects the AP excitation wave due to the intensity and direction of the externally applied deformation. In addition, this behavior does not correspond to a mere linear mapping from the reference to the deformed configuration (as a visual scaling of the image would easily show), but one observes that mechanical deformations induce higher, nonlinear and non-trivial anisotropies and heterogeneities in the tissue.

**Figure 2:**
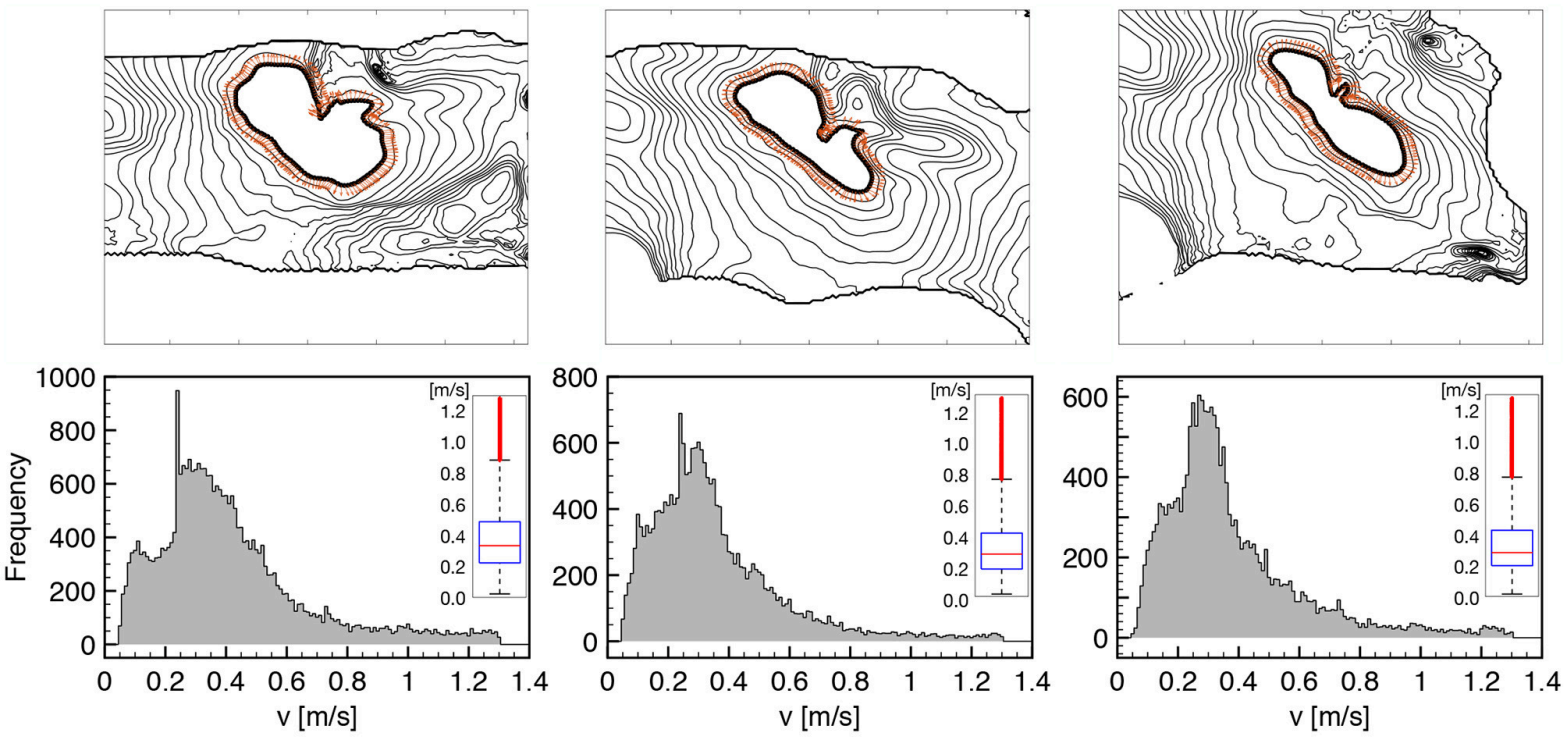
CV histograms measured on tissue wedges for three different loading states overlapping local measures for seven consecutive activations at constant pacing cycle length of 1s. All the normal directions to the AP propagation are considered as indicated by orange arrows on a representative isochrone contour. The box plot of the distribution is provided as inset for the three histogram, respectively, highlighting the amount of dispersion and the reduction of CV under stretch (see Table 1 for details). Cut-off of spurious values is set at 0.05 and 1.3 *m*/*s*.

To better characterize such features, in Figure 2 we provide an extended analysis of the local conduction velocity (CV) thorough histogram plots measured as follows:

- we identify wavefront isochrones at 50% of depolarization for eleven consecutive frames at 2ms each (this produces ten consecutive measures of CV per direction selected);
- we compute the contour normal direction and the corresponding distance between consecutive isochrones;
- we measure the local CV for all the computed normal directions, along the isochrone path and for seven consecutive action potential activations at constant pacing cycle length of 1s;
- we exclude the extreme values from the histogram to take out spurious results, e.g., boundary effects.

The chosen methodology allows to represent tissue heterogeneity, provides a robust measure of the local CV distribution characterizing the underlying ventricular structure, and homogenizes physiological beat-to-beat variabilities. We summarize the results of such an extended analysis in Table 1, distinguishing between the three loading cases as described in Figure 1, providing sample size and statistical features of the computed CV histogram distribution, i.e., mean and median. We also provide the box plot representation of the obtained distributions for the three stretch states, respectively, to further highlight dispersion of the measured velocities. Every single feature in the study confirms a slower conduction velocity under stretch, and this behavior is full agreement with previous studies (Ravelli, 2003).

**Table 1:** Summary of the local CV measurement, indicating histogram sample size and representative statistical features of the computed distribution: mean and median.

	**No-Stretch**	**Horizontal stretch**	**Vertical stretch**
Sample size	28,760	20,645	18,746
Mean [m/s]	0.42	0.36	0.38
Median [m/s]	0.36	0.31	0.32

**Figure 3:**
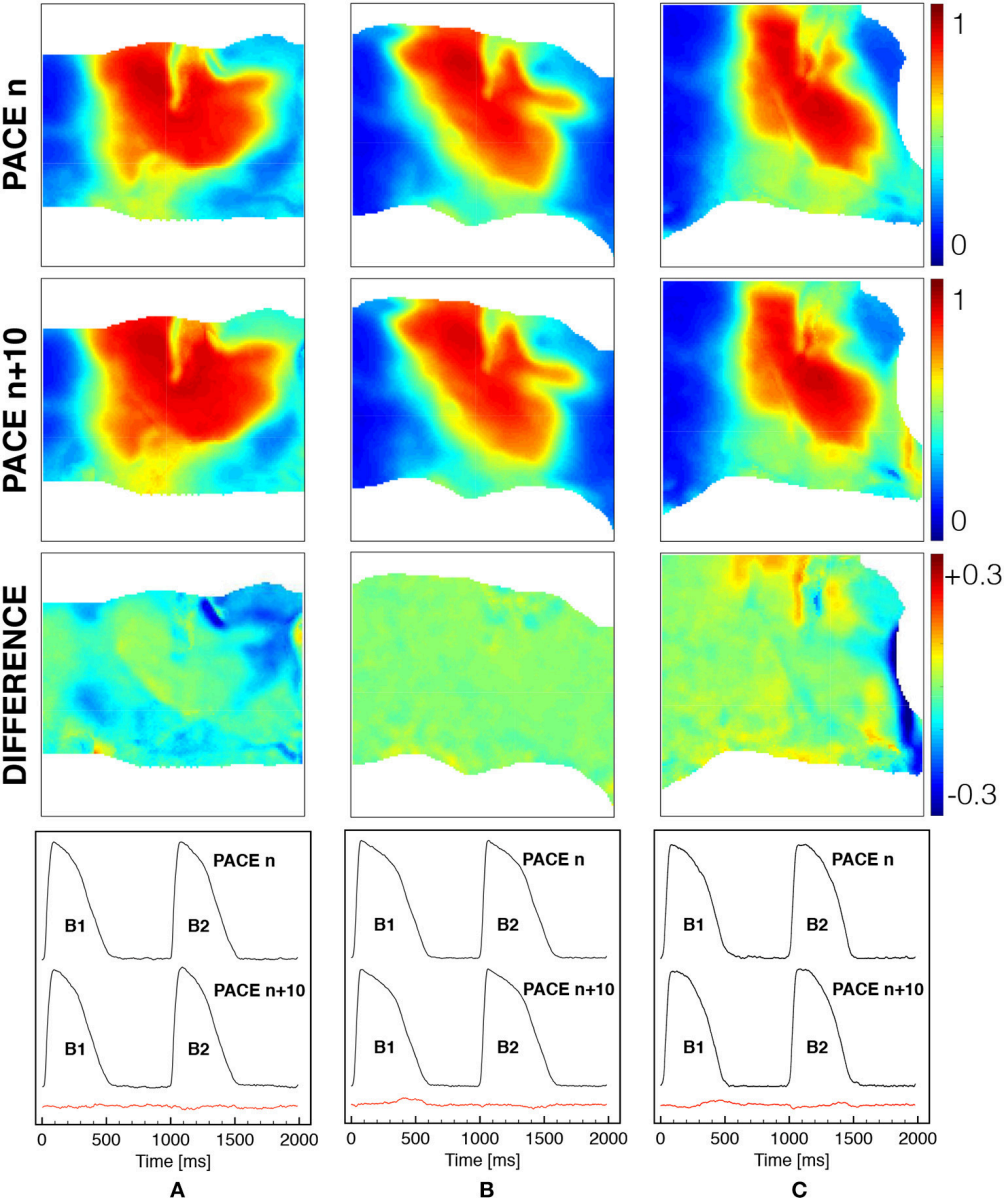
Spatial and temporal comparison of ventricular activation at constant pacing cycle length of 1s under different mechanical loadings [free **(A)**, horizontal **(B)** and vertical **(C)** stretch as in Figure 1]. The first two rows show the spatial distribution of the normalized voltage for beat *n* and beat *n* + 10 with the corresponding difference in the third row (color code is indicated). The last row indicates the time course of a representative pixel in the center of the field of view for two consecutive beats *n* and *n* + 10 with the corresponding difference provided in the red trace.

Also, in Figure 3 we demonstrate that the tissue is at steady-state for the selected stimulation rate providing a quantitative comparison of the spatial and temporal activation sequences. In particular, after several activations (>5), beat *n* and beat *n* + 10 are shown for a selected frame in terms of normalized AP distribution and its spatial difference, as well as comparing the time course of two consecutive activations (B1, B2) for a representative pixel under the field of view. In both cases, the spatio-temporal differences recorded are within the physiological variability of a ventricular wedge, and the tissue shows a steady-state regime which is considered at resting state for the numerical model.

Clear MEF effects evidenced in the previous experimental exercise suggest the incorporation of deformation and stress into the conduction properties of the cardiac tissue itself. The preliminary character of the proposed minimal model implies that we do not take into account the intrinsic structural variability of the tissue, but we stress that these effects will be investigated in future validation works. Accordingly, as a base line model, in the present study we will adapt the formulation recently proposed in Cherubini et al. (2017) and designed for general purpose stress-diffusion couplings. Doing so will allow us to readily and selectively incorporate two main MEF-related mechanisms into the computational modeling of cardiac electromechanics: (i) stretch-activated currents (SAC) and (ii) stress-assisted diffusion (SAD). The first *paradigm* relates the deformed mechanical state to the excitability of the medium via additional reaction functions (ionic-like currents); whereas the second one collects the homogenized effects of the deformation field on the diffusion processes originating the spatio-temporal patterns of the membrane voltage.

Within such a framework, we expect stretch-activated currents and stress-assisted diffusion to counterbalance each other by locally enhancing tissue excitability as well as smoothing the excitation wave according to the mechanical state of the tissue. In particular, since an external loading activates SAC at locations where the stretch is high and, at the same time, induces an heterogeneous and anisotropic diffusion tensor via the SAD mechanisms, our study focuses on the role of different mechanical boundary conditions in affecting action potential propagation and onset of arrhythmias. Accordingly, these two MEF mechanisms will be studied numerically in terms of three basic lines. First, by conducting a parametric analysis of the competing nonlinearities such to identify the limits of applicability of the proposed models. In particular, we select in the SAD mechanisms the most reliable modeling approach able to reproduce the experienced conduction velocity reduction upon an applied static loading state. Then, by performing a selective investigation of spiral onset protocols we will characterize the additional nonlinearities that arise due to MEF. Here we identify the different time span of the vulnerable window obtained via an S1S2 excitation protocol. Finally, by means of long-run analyses of arrhythmic scenarios, we compare and contrast static and dynamic displacement and traction loadings on a two-dimensional, idealized tissue slab. In this regard, we show how spiral core meandering results highly affected by the mechanical state and becomes unstable when SAC and SAD parameters are stronger.

Our results highlight several interesting conclusions regarding the propagation of the excitation wave in the presence of two competing MEF effects. These findings call for novel and additional experimental investigations. Finally, we provide a thorough discussion of the applicability of the proposed modeling approach and its extensions toward more realistic and multiphysics scenarios.

## 2. Methods

The classical stress-assisted formulation proposed in Aifantis (1980) was developed in the context of dilute solutes in a solid. A similarity exists between this fundamental process and the propagation of membrane voltage within cardiac tissue. Indeed, on a macroscopically rigid matrix, the propagating membrane voltage can be regarded as a continuum field undergoing slow diffusion. Here we consider a similar approach (developed in Cherubini et al., 2017) which generalizes Fick's diffusion by using the classical Euler's axioms of continuously distributed matter. In particular, the balance of momentum can be imposed such to ensure frame invariance, a property of high importance in mechanical applications (Tadmor et al., 2012). We also assume quasi-static conditions for the continuum body, such that its macroscopic response is, in principle, independent from the diffusion process. On the contrary, the diffusion process will strongly depend on the mechanical state of the tissue.

### 2.1. Continuum electromechanical model

We will assume that the body is a hyperelastic material and its motion will be described using finite kinematics. We will adopt an indicial notation where repeated indices indicate summation. We identify the relationship between material (reference), *X*_*I*_, and spatial (deformed), *x*_*i*_, coordinates via the smooth map *x*_*i*_(*X*_*I*_). The deformation gradient tensor *F*_*iI*_ = ∂*x*_*i*_/∂*X*_*I*_ allows to determine further properties of the continuum's motion. We indicate with *J* = det*F*_*iI*_ the Jacobian of the map and with *C*_*IJ*_ = *F*_*kI*_*F*_*kJ*_ and *B*_*ij*_ = *F*_*iK*_*F*_*jK*_ the right and left Cauchy-Green deformation tensors, respectively. We assume that the generic myocardial fiber direction (the unit vector characterizing the microstructural property of the continuum body) in the material configuration, *a*_*I*_, is mapped to the deformed configuration as *a*_*i*_ = *F*_*iJ*_*a*_*J*_ such that we can define the current fiber *a*_*i*_ = *a*_*I*_/λ. Following the standard frame indifference mechanical framework (Spencer, 1989), these quantities are related to the invariants of the deformation in the following manner

(1) $$\begin{array}{l} I_{1}=C_{II},I_{2}=\frac{1}{2}\left[{\left(C_{II}\right)}^{2}-C_{IJ}C_{JI}\right],I_{3}=\det C_{IJ}=J^{2},\\ \;I_{4}=C_{IJ}a_{I}a_{J}. \end{array}$$

The principal invariants *I*_1_ and *I*_2_ rule the deviatoric response of the medium, the third invariant *I*_3_ quantifies volumetric changes of the material, while the fourth pseudo-invariant *I*_4_ measures the directional *fiber stretch*, λ. This last entity is intrinsically directional, so for two-dimensional models, we will simply assign a horizontal myocardial direction (1, 0)^*T*^. In what follows, the symbol δ_*ij*_ denotes the second-order identity tensor.

As anticipated above, we will base our model on the stress-assisted diffusion formulation from Cherubini et al. (2017). We do however, generalize the governing equations adopting a more accurate nondimensional three-variable model of cardiac action potential (AP) propagation introduced in Fenton and Karma (1998b), and we will account for SAC (Panfilov and Keldermann, 2005), that were not considered in Cherubini et al. (2017). Even though several more physiological assumptions could be made, here we will focus on a purely phenomenological approach.

In the deformed configuration, the electrophysiological model consists of three variables: the membrane potential *u*, and a fast and slow transmembrane ionic gates *v, w*. They satisfy the following RD system

(2a) $$\begin{array}{l} \frac{\partial u}{\partial t}=\frac{\partial }{\partial x_{i}}\left(d_{ij}\left(\sigma_{ij}\right)\frac{\partial u}{\partial x_{j}}\right)-{\text{I}}_{\text{ion}}\left(u,v,w\right)+\text{Isac}\left(\lambda ,u\right)+{\text{I}}_{\text{ext}}, \end{array}$$

(2b) $$\begin{array}{l} \frac{dv}{dt}=\left(1-H_{c}\right)\left(\frac{1-v}{\tau_{v}^{-}}\right)-H_{c}\frac{v}{\tau_{v}^{+}}, \end{array}$$

(2c) $$\begin{array}{l} \frac{dw}{dt}=\left(1-H_{c}\right)\left(\frac{1-w}{\tau_{w}^{-}}\right)-H_{c}\frac{w}{\tau_{w}^{+}}, \end{array}$$

where Neumann zero-flux boundary conditions are imposed for Equation (1a), i.e., [*d*_*ij*_∂*u*/∂*x*_*j*_]*n*_*i*_ = 0, where *n*_*i*_ is the outward normal on the domain boundary. System (1) describes the propagation of a normalized dimensionless membrane potential, which can be mapped to physical quantities as *u* = (*V*_*m*_−*V*_*o*_)/(*V*_*fi*_−*V*_*o*_) (see Fenton and Karma, 1998b for details as modified Beeler-Reuter fit) where *V*_*m*_ stands for the physical transmembrane potential, *V*_*o*_ is the resting membrane potential and *V*_*fi*_ represents the Nernst potential of the fast inward current. In Equation (1a), the total transmembrane density current, I_ion_(*u, v, w*), is the sum of a fast inward depolarizing current, I_*fi*_(*u, v*), a slow rectifying outward current, I_*so*_(*u*), and a slow inward current, I_*si*_(*u, w*), given by

$$\begin{array}{l} {\text{I}}_{fi}\left(u,v\right)=-\frac{v}{\tau_{d}}H_{c}\left(1-u\right)\left(u-u_{c}\right),\\ {\text{I}}_{so}\left(u\right)=\frac{u}{\tau_{o}}\left(1-H_{c}\right)+\frac{1}{\tau_{r}}H_{c},\\ {\text{I}}_{si}\left(u,w\right)=-\frac{w}{2\tau_{si}}\left(1+\tanh\left[k\left(u-u_{c}^{si}\right)\right]\right), \end{array}$$

where $\tau_{v}^{-}\left(u\right)=H_{v}\tau_{v1}^{-}+\left(1-H_{v}\right)\tau_{v2}^{-}$ is the time constant governing the reactivation of the fast inward current, and *H*_*x*_ = *H*_*x*_(*u*−*u*_*x*_) is the standard Heaviside step function. I_ext_ is the space and time-dependent external stimulation current with amplitude ${\text{I}}_{\text{ext}}^{\max}$. All model parameters are collected in Table 2.

**Table 2:** Model parameters for the electromechanical three-variable model, considered as in Fenton and Karma (1998b) and Cherubini et al. (2017).

ḡ_*fi*_	4	τ_*d*_	*C*_*m*_/ḡ_*fi*_	$\tau_{w}^{+}$	667	ϵ_0_	0.1	*u*^init^ = 0
τ_*r*_	50	*C*_*m*_	1 μF/cm^2^	$\tau_{w}^{-}$	11	*k*_*Ta*_	9.58	*v*^init^ = 1
τ_*si*_	45	*V*_*o*_	−85	*u*_*c*_	0.13	*c*_1_	6	*w*^init^ = 1
τ_*o*_	8.3	*V*_*fi*_	15	*u*_*v*_	0.055	*c*_2_	2	φ^init^ = 0
$\tau_{v}^{+}$	3.33	*D*_0_	1·10^−3^	$u_{c}^{si}$	0.85	*G*_*s*_	[0;0.25]	*p*^init^ = 0
$\tau_{v1}^{-}$	1000	*D*_1_	[−1.5;0]·10^−4^	*k*	10	*u*sac	0.4	$T_{a}^{\text{init}}=0.2$
$\tau_{v2}^{-}$	19.6	*D*_2_	1·10^−5^	$I_{ext}^{\max}$	2	*t*_max_	9	

*Time units are ms, length is cm, the term ḡ_fi_ is in mS/cm^2^, dimensional voltages are in mV, and stiffness in MPa. Square brackets indicate range of parameter variability, and the rightmost column specifies initial conditions for a resting tissue*.

The mechanical problem, stated also on the current configuration and occupying the domain Ω(*t*), respects the balance of linear momentum and mass, written in terms of displacement, φ, and pressure, *p*, and set in a quasi-static form. The problem is complemented with displacement and traction boundary conditions set on two different parts of the boundary Γ_*D*_ or Γ_*N*_:

(3a) $$\begin{array}{l} \frac{\partial \sigma_{ij}}{\partial x_{i}}=0\text{ and }\rho d\hat{v}=\rho_{0}d\hat{V},\text{ in }\Omega (t), \end{array}$$

(3b) $$\begin{array}{l} \;\varphi =\tilde{\varphi }(t),\text{ on }\Gamma_{D}(t), \end{array}$$

(3c) $$\begin{array}{l} \;\sigma_{ik}n_{k}={\tilde{t}}_{i}(t),\text{ on }\Gamma_{N}(t), \end{array}$$

where ρ_0_, ρ and $d\hat{V},d\hat{v}$ are the densities and volumes of the solid in the undeformed and deformed configurations, respectively. In Equation (3b), $\tilde{\varphi }\left(t\right)$ is a known (possibly time-dependent) displacement and in Equation (3c), ${\tilde{t}}_{i}\left(t\right)$ is a (possibly time-dependent) traction force. In both cases, the tissue is stretched up to a maximum level of 20% of the resting length such to activate all MEF components. In addition, the time-variation of the imposed boundary conditions is much slower than the governing dynamic physical processes, and therefore a quasi-static mechanical equilibrium is maintained.

The two sub-problems (Equations 2, 3) are completed via the following mixed constitutive prescriptions for incompressible isotropic hyperelastic materials (*J* = 1):

(4a) $$\begin{array}{l} \sigma_{ij}=2c_{1}B_{ij}-2c_{2}B_{ij}^{-1}-p\delta_{ij}+T_{a}\delta_{ij}, \end{array}$$

(4b) $$\begin{array}{l} \frac{\partial T_{a}}{\partial t}=\epsilon (u)(k_{T_{a}}u-T_{a}), \end{array}$$

(4c) $$\begin{array}{l} d_{ij}(\sigma_{ij})=D_{0}\delta_{ij}+D_{1}\sigma_{ij}+D_{2}\sigma_{ik}\sigma_{kj}, \end{array}$$

(4d) $$\begin{array}{l} {\text{I}}_{\text{sac}}\left(\lambda ,u\right)=G_{s}H_{\text{sac}}(\lambda -1)(u_{\text{sac}}-u). \end{array}$$

Equation (4a) specifies a constitutive form for the Cauchy stress tensor (total equilibrium stress in the current deformed configuration) highlighting two multiscale contributions on the tissue deformation. First, the passive material response follows that of an incompressible Mooney-Rivlin hyperelastic solid and it is characterized by two stiffness parameters *c*_1_ and *c*_2_; and secondly, the active component contributing to the total stress in the form of an additional hydrostatic force with amplitude *T*_*a*_. The dynamics of *T*_*a*_ are described by Equation (4a), where the constant *k*_*Ta*_ modulates the amplitude of the active stress contribution, while ϵ(*u*) is a contraction switch function: ϵ(*u*) = ϵ_0_ if *u* < 0.005, and ϵ(*u*) = 10ϵ_0_ if *u* ≥ 0.005.

Equation (4c) characterizes the stress-assisted diffusion contribution describing the effect of tissue deformation on the AP spreading. The parameter *D*_0_ represents the usual diffusion coefficient for isotropic media, i.e., diffusivity = [L^2^ T^−1^], while *D*_1_ and *D*_2_ introduce the impact of mechanical stress through linear and nonlinear contributions, respectively, on the diffusive flux. Accordingly, *D*_1_ and *D*_2_ have units of [L^2^ T^−1^ P^−1^] and [L^2^ T^−1^ P^−2^], respectively. We also remark that Equation (4c) reduces to the characterization of the classical diffusion equation for *D*_1_ ≡ *D*_2_ = 0.

Finally, Equation (4d) describes the stretch-activated current contribution (which is usually adopted as the sole MEF effect). The term Isac(λ, *u*) affects the ionic (reaction) currents in the electrophysiological system and is formulated as a linear function of the membrane potential *u* and the fiber stretch λ. Here, *G*_*s*_ modulates the amplitude of the current, *u*sac represents a referential (resting) potential while, *H*_sac_ is a switch activating this additional reaction current only when the myocardial fiber is elongated, i.e., *H*_sac_ = 1 for λ ≥ 1 and *H*_sac_ = 0 for λ < 1.

We also introduce the definition of spiral tip (core of the spiral wave) as the point with instantaneous null velocity (see Fenton and Karma, 1998b for details). In practice, for two-dimensional domains, we choose an isopotential line of constant membrane voltage, *u*(*R*_*I*_, *t*) = *u*_iso_, where *R*_*I*_ = *x*_tip_*X*_*I*_ + *y*_tip_*Y*_*I*_ represents the position vector in the reference undeformed configuration identifying the boundary between depolarized and repolarized regions. Accordingly, the spiral tip can be defined as the point in space where the excitation front meets the repolarization waveback of the action potential, conforming with the operative definition:

(5) $$\begin{array}{l} u\left(R_{I},t\right)-u_{\text{iso}}=\frac{\partial u\left(R_{I},t\right)}{\partial t}\equiv 0. \end{array}$$

We numerically identify the tip coordinates (*x*_tip_, *y*_tip_) by considering *u*_iso_ = 0.5 with tolerance of 10^−4^.

### 2.2. Numerical approximation

The electromechanical problem is rewritten in the undeformed configuration and subsequently computationally solved via a finite element method. Even if the model originates as an extension of our contribution in Cherubini et al. (2017), the numerical method employed here is simpler, as we do not solve for stresses explicitly but rather postprocess them from the computed discrete displacements. The overall numerical scheme for active stress electromechanics with SAC is therefore not precisely novel, but we will still provide a few details for sake of completeness of the presentation and future reproducibility of results. Further details could be found in e.g., Ruiz-Baier (2015). We discretize displacements with vectorial piecewise quadratic and continuous polynomials, and the pressure field using piecewise linear and discontinuous elements. All remaining unknowns (associated to the electrophysiology and to the active tension) are approximated using piecewise linear and continuous elements. Let us then consider a regular, quasi-uniform partition $T_{h}$ of $\bar{\Omega \left(0\right)}$ into triangles *T* of diameter *h*_*T*_, where $h=\max\left\{h_{T}:T\in T_{h}\right\}$ is the meshsize. The finite element spaces mentioned above are defined as (see e.g., Quarteroni and Valli, 1994)

$$\begin{array}{l} {\text{H}}_{h}:\;=\{\psi \in {\text{H}}^{1}(\Omega (0)):\;\psi {|}_{T}\in {\left[{\mathbb{P}}_{2}(T)\right]}^{2}\forall T\in T_{h},\text{ and}\\ \psi =0\text{ on }\Gamma_{D}(0)\},\\ Q_{h}:\;=\{q\in L^{2}(\Omega (0)):q{|}_{T}\in {\mathbb{P}}_{1}(T)\forall T\in T_{h}\},\\ W_{h}:\;=\{\psi \in H^{1}(\Omega (0)):\psi {|}_{T}\in {\mathbb{P}}_{1}(T)\forall T\in T_{h}\}, \end{array}$$

for the case of clamped boundaries at Γ_*D*_(0).

Let us also construct an equispaced partition of the time domain $0=t^{0}<t^{1}=\Delta t<\cdots <t^{M}=t_{\max}$. The coupled problem is solved sequentially between the mechanical and electrochemical blocks. A description of the needed computations at each time step *t*^*n*^ is as follows:

**Step 1:** From the known values $u_{h}^{n},v_{h}^{n},w_{h}^{n},T_{a,h}^{n},D_{h}^{n},\lambda_{h}^{n}$, find $u_{h}^{n+1},v_{h}^{n+1},w_{h}^{n+1},T_{a,h}^{n+1}$ such that

$$\begin{array}{l} \int_{\Omega \left(0\right)}\frac{u_{h}^{n+1}}{\Delta t}\psi_{h}^{u}+\int_{\Omega \left(0\right)}D_{h}^{n}\nabla u_{h}^{n+1}\cdot \nabla \psi_{h}^{u}\\ \;=\int_{\Omega \left(0\right)}\left[\frac{u_{h}^{n}}{\Delta t}+{\text{I}}_{\text{ion}}\left(u_{h}^{n},v_{h}^{n},w_{h}^{n}\right)+{\text{I}}_{\text{sac}}\left(\lambda_{h}^{n},u_{h}^{n}\right)+{\text{I}}_{\text{ext}}\right]\psi_{h}^{u},\\ \frac{1}{\Delta t}\int_{\Omega \left(0\right)}v_{h}^{n+1}\psi_{h}^{v}=\int_{\Omega \left(0\right)}\left[\frac{1}{\Delta t}v_{h}^{n}+f_{v}\left(u_{h}^{n},v_{h}^{n}\right)\right]\psi_{h}^{v},\\ \frac{1}{\Delta t}\int_{\Omega \left(0\right)}w_{h}^{n+1}\psi_{h}^{w}=\int_{\Omega \left(0\right)}\left[\frac{1}{\Delta t}w_{h}^{n}+f_{w}\left(u_{h}^{n},w_{h}^{n}\right)\right]\psi_{h}^{w},\\ \frac{1}{\Delta t}\int_{\Omega \left(0\right)}T_{a,h}^{n+1}\psi_{h}^{T_{a}}=\int_{\Omega \left(0\right)}\left[\frac{1}{\Delta t}T_{a,h}^{n}+f_{T_{a}}\left(u_{h}^{n},T_{a,h}^{n}\right)\right]\psi_{h}^{T_{a}}, \end{array}$$

for all $\left(\psi_{h}^{u},\psi_{h}^{v},\psi_{h}^{w},\psi_{h}^{T_{a}}\right)\in {\left[V_{h}\right]}^{4}$. This scheme for the electric/activation system is given in a first-order semi-implicit form: the nonlinear reaction terms and the coupling stress-assisted diffusion are taken explicitly, while the linear part of diffusion is advanced implicitly. Here

$$\begin{array}{l} D_{h}^{n}=D_{0}{\text{C}}^{-1}\left(\varphi_{h}^{n}\right)+\frac{D_{1}}{J\left(\varphi_{h}^{n}\right)}\text{S}\left(\varphi_{h}^{n}\right)+\frac{D_{1}}{J{\left(\varphi_{h}^{n}\right)}^{2}}\text{S}{\left(\varphi_{h}^{n}\right)}^{2},\\ \lambda_{h}^{n}=\sqrt{C_{11}\left(\varphi_{h}^{n}\right)}, \end{array}$$

are the explicit approximation of the stress-assisted diffusivity and of the stretch in the fiber direction, all in the reference configuration.

**Step 2:** Given the activation value $T_{a,h}^{n+1}$ computed in Step 1 of this iteration, solve the nonlinear elasticity equations

$$\begin{array}{ll} \int_{\Omega \left(0\right)}\text{F}\left(\varphi_{h}^{n+1}\right)\text{S}\left(\varphi_{h}^{n+1},p_{h}^{n+1},T_{a,h}^{n+1}\right):\nabla \psi_{h}=0 & \;\forall \psi_{h}\in {\text{H}}_{h},\\ \int_{\Omega \left(0\right)}q_{h}\left[J\left(\varphi_{h}^{n+1}\right)-1\right]=0 & \;\forall q_{h}\in Q_{h}, \end{array}$$

where

$$\begin{array}{l} \text{S}=2\left[c_{1}+c_{2}\text{tr}\left(\text{C}\left(\varphi_{h}^{n+1}\right)\right)\right]\text{I}-2c_{2}\text{C}\left(\varphi_{h}^{n+1}\right)\\ \;-p_{h}^{n+1}J\left(\varphi_{h}^{n+1}\right){\text{C}}^{-1}\left(\varphi_{h}^{n+1}\right)+T_{a,h}^{n+1}{\text{C}}^{-1}\left(\varphi_{h}^{n+1}\right), \end{array}$$

is the second Piola-Kirchhoff stress tensor.

**Step 3:** The solution of the problem in Step 2 uses a Newton-Raphson method whose iterations are terminated once the energy residual drops below the relative tolerance of 10^−6^. The solution to each linear tangent problem is conducted with the BiCGSTAB method preconditioned with an incomplete LU(0) factorization. The iterations of the Krylov solver are terminated after reaching the absolute tolerance 10^−5^. The residual computation for the mechanical problem also contains the terms arising from time-dependent displacement or traction boundary conditions, which also need to be assigned at each timestep. For instance, in an uniaxial test (denoted dynamic displacement in the examples below), the left segment of the boundary is clamped (zero displacements are imposed), the bottom and top edges are subject to zero normal stress, and the right edge is pulled according to the displacement $\tilde{\varphi }\left(t\right)={\left[0.2L\sin^{2}\left(\pi /400t\right),0\right]}^{T}$.

**Figure 4:**
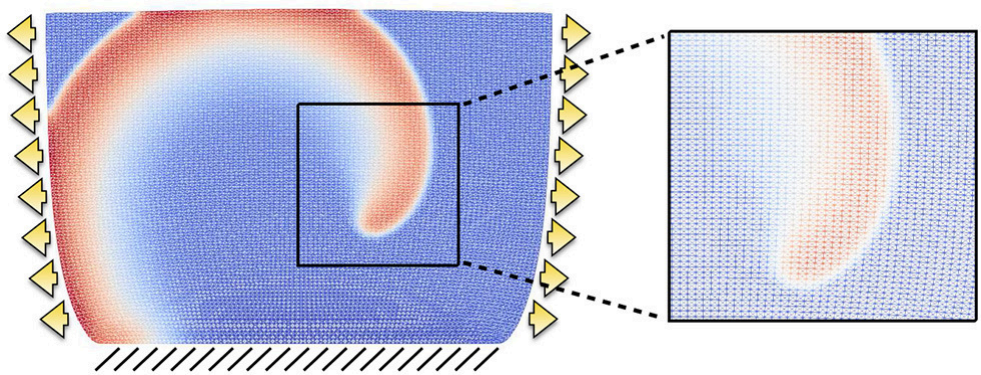
Example of structured mesh employed in the computational results. The grid is displayed on the deformed configuration when the domain is subject to traction (arrows) and fixed displacement (lines) boundary conditions, and a zoom exemplifies the mesh resolution for a rather coarse spiral front.

All tests are conducted using a two-dimensional slab of dimensions *L* × *L* = 6.2 × 6.2cm^2^, which is the same configuration used to produce the dynamics analyzed in Fenton and Karma (1998b). The computational domain is discretized with a structured triangular mesh of 10,000 elements. After a mesh convergence test involving conduction velocities and reproducing the expected values for planar excitation waves reported in Fenton and Karma (1998b), we proceeded to fix the temporal and spatial resolutions to Δ*t* = 0.1ms, *h* = 0.062cm, respectively. A representative example of the mesh is provided in Figure 4, plotted in the deformed configuration under both traction and displacement boundary conditions and highlighting the spiral wave resolution. All numerical tests were carried out using the open-source finite element library FEniCS (Alnæs et al., 2015).

## 3. Results

In the following, we adopt a parametric setup fitted for the modified Beeler-Reuter model (Equation 2), while selectively changing MEF parameters (*D*_1_, *G*_*s*_). This choice provides a reference, unloaded, model configuration with constant CV of 0.42 m/s and a circular meandering for a free spiral on a homogeneous and isotropic domain. Such values deviate as the MEF coupling is activated.

### 3.1. Conduction velocity analysis

We start analyzing the parameter space associated to the two MEF contributions in our model. That is, the stress-assisted coefficients *D*_1_, *D*_2_ and the SAC amplitude *G*_*s*_. The study will be restricted to a static homogeneous stretched state (e.g., a uniaxial Dirichlet boundary condition **φ** = [0.2*L*, 0]^*T*^ set on the right edge of the domain). All remaining material and electrophysiology parameters will be kept constant, except that we fix the relative influence of the nonlinear contribution in the stress-assisted diffusion, by setting *D*_2_ to be one order of magnitude smaller than *D*_1_. This configuration will highlight MEF effects in a minimal, but still comprehensive manner.

**Figure 5:**
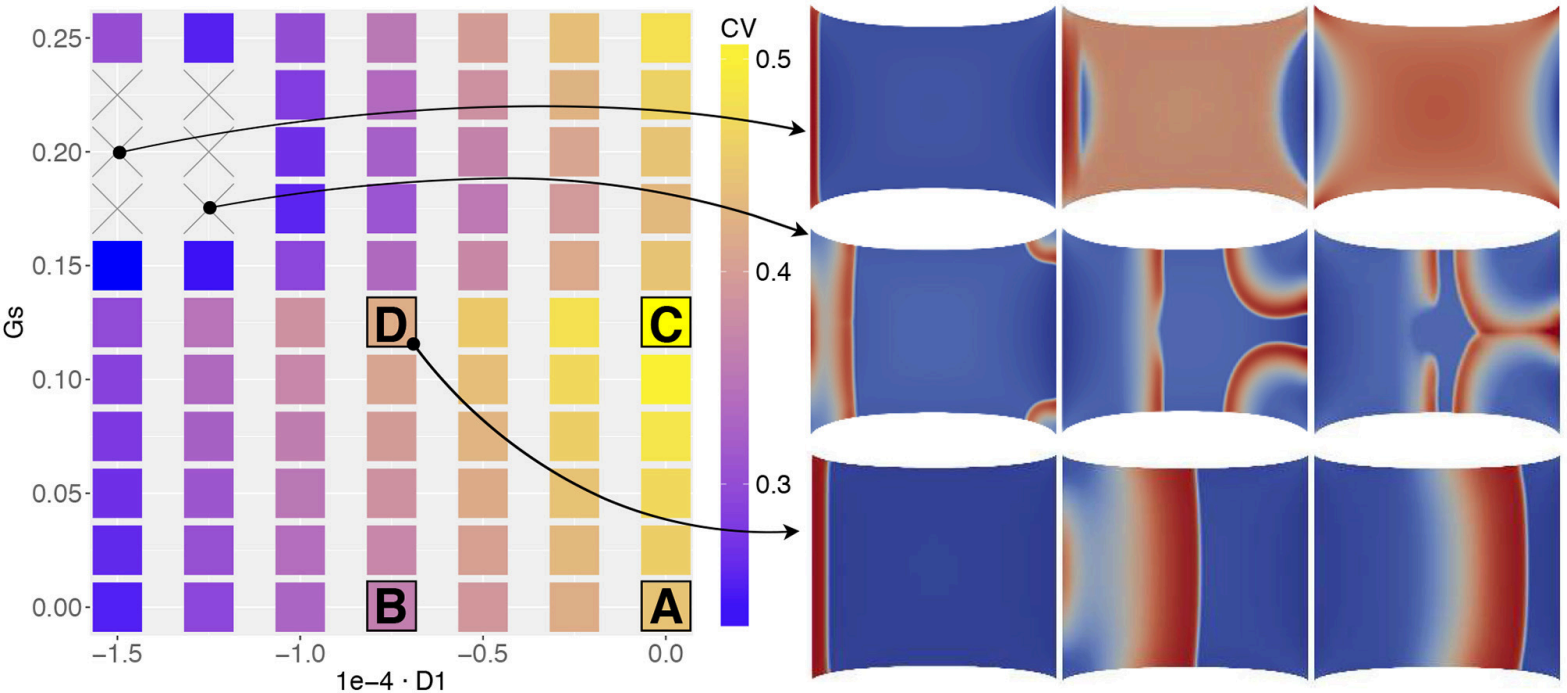
MEF parameter space associated to the conduction velocity measured on the propagating front of a planar excitation wave (stimulation on the left edge and propagation toward the right boundary) elicited on a static uniaxially stretched domain (CV in [*m*/*s*]). Four selected combinations of MEF parameters **(A,B,C,D**, in Table 3) are highlighted together with two additional cases in which CV was not recorded. On the right, three consecutive time frames of the activation are selected.

Figure 5 portrays the conduction velocity obtained for all combinations of (*D*_1_, *G*_*s*_) on the parameter space. The quantity is measured as the wave-front velocity of a planar excitation wave along its propagation. The plot illustrates the variability of the recorded CV amplitude (in the range 0.25–0.5 m/s) according to the MEF coupling intensity variation and to histogram measures in Figure 2. In particular, starting from a physiological baseline of 0.42 m/s, when neither SAC nor SAD is present (*D*_1_ = 0, *G*_*s*_ = 0), we observe a net increase of CV for (*D*_1_ = 0, *G*_*s*_ > 0) while we recover CV decrements for (*D*_1_ < 0, *G*_*s*_ = 0). This specific aspect reproduces what is expected from experimental evidence, i.e., MEF decreases the CV of the excitation wave (Ravelli, 2003).

**Figure 6:**
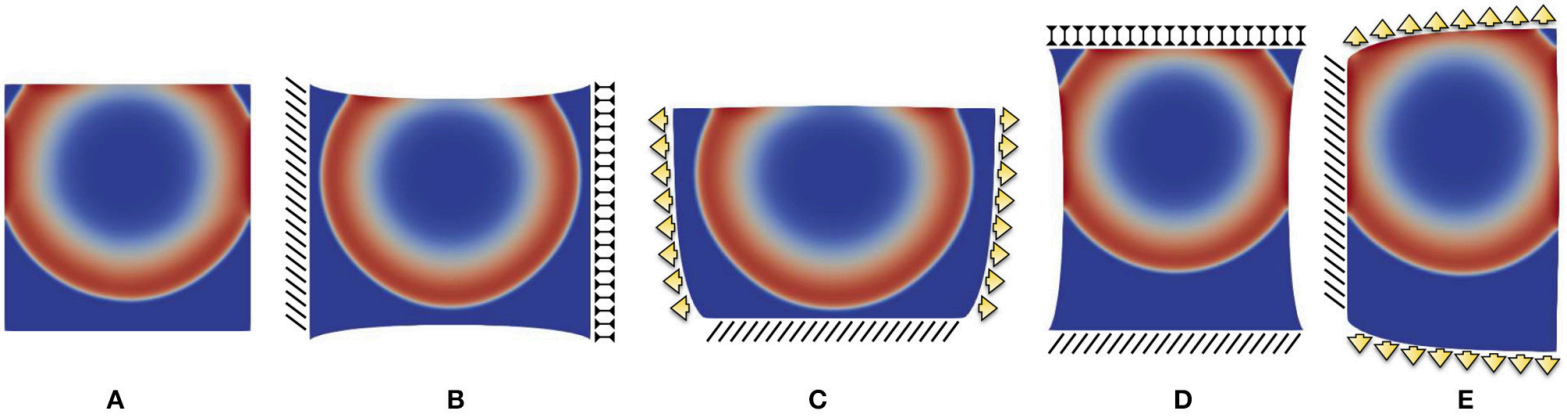
Point-wise activation frame for five different static boundary conditions qualitatively reproducing ventricle wedge preparation measurements considering the parameter combination $\left(D_{1},G_{s}\right)=\left(-0.75\cdot 10^{-4},0\right)$: **(A)** free edges, **(B)** horizontal displacement, **(C)** vertical displacement, **(D)** horizontal traction, **(E)** vertical traction. Color code refers to the normalized action potential.

Besides, for higher values of *G*_*s*_, we obtain two unexpected results. First, for *G*_*s*_ > 0.15 we observe a decrement of CV for different values of *D*_1_. Second, for the particular combination $\left(D_{1}<-10^{-4},G_{s}>0.15\right)$ the wave disappears from the domain or annihilates due to excessive activation (see e.g., side panels in Figure 5 or the top row in **Figure 8**). Consequently, we are not able to measure any propagation (which reflects in the combinations with × of the figure). This last result is somehow counterintuitive since, as evidenced by Figure 1, we experimentally experience a complete depolarization of the tissue with AP propagation, in the case of fixed stretch. To support this point, in Figure 6 we provide a representative sequence of point-wise activations delivered on our simplified 2D domain and mimicking the experimental protocol conducted in Figure 1 for a selected parameter choice, i.e., $\left(D_{1},G_{s}\right)=\left(-0.75\cdot 10^{-4},0\right)$. In this case, the AP excitation wave propagates differently according to the applied stretch state, both horizontal and vertical displacement and traction. In addition, the computed CVs change similarly to what observed in Figure 2. We remark that such a comparison with experimental observations is purely qualitative and does not represent a definitive validation of the model.

### 3.2. S1-S2 excitation protocol

We further investigate the strength of MEF coupling effects. In particular, we want to determine which specific contribution (stretch-activated currents or stress-assisted diffusion) exhibits a better match against experimental evidence, and for this we assess changes in the S1-S2 stimulation protocol. In practice, in order to induce a spiral wave on an excitable tissue, one typically generates a planar electrical excitation (S1), followed by a second broken stimulus (S2) during the repolarization phase of the S1 wave, the so called vulnerable window (Karma, 2013). In our case, we selected a reduced set of MEF parameters (*D*_1_, *G*_*s*_) indicated in Table 3 as A,B,C,D. These values are motivated by the results from Figure 5. In particular, we select only the parameter combinations that produce either a unique decrement or increment of CV.

**Table 3:** Parameter calibration associated to the S1-S2 protocol.

	***D*_1_**	***G*_*s*_**	**CV [m/s]**	**$t_{{\text{S}}_{2}}^{\text{min}}-t_{{\text{S}}_{2}}^{\text{max}}$ [ms]**
A:	0	0	0.45	225–240
B:	−0.75·10^−4^	0	0.36	243–255
C:	0	0.125	0.52	133–147
D:	−0.75·10^−4^	0.125	0.42	143–157

*Combination of MEF parameters (D_1_, G_s_), corresponding CV, minimum, $t_{{\text{S}}_{2}}^{\text{min}}$, and maximum, $t_{{\text{S}}_{2}}^{\text{max}}$, stimulation time required for spiral wave onset (vulnerable window)*.

**Figure 7:**
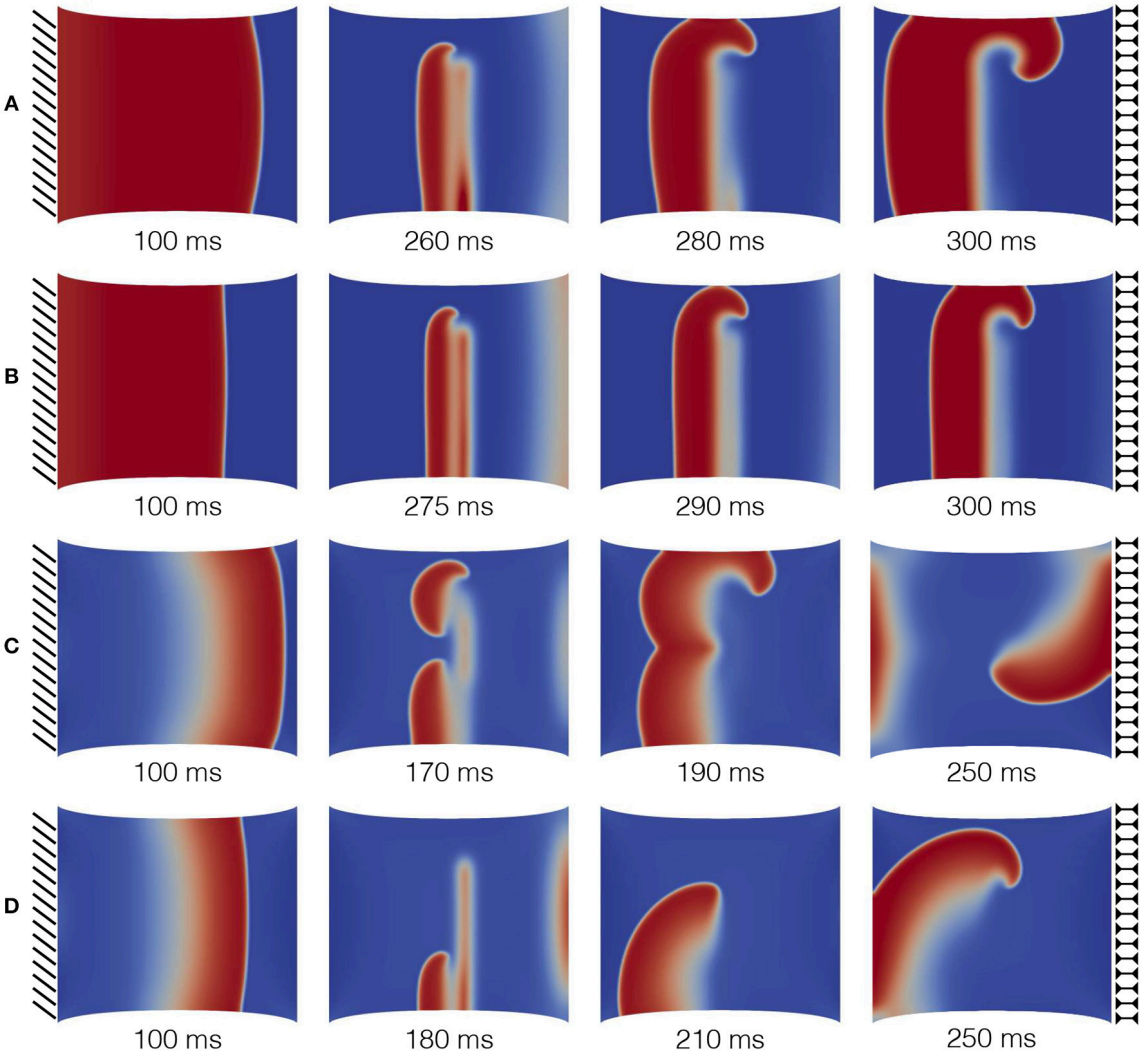
S1-S2 stimulation protocol applied on a static uniaxial stretched configuration for different combinations of MEF parameters (*D*_1_, *G*_*s*_) as provided in Table 3. The color code refers to normalized dimensionless membrane potential, *u*, (blue-red mapped to [0–1]). Selected time frames are provided in the subpanels.

Figure 7 shows the different dynamics obtained via the S1-S2 protocol for the four different sets of MEF parameters. The first column is set at 100ms from the S1 stimulus for all the combinations, while the remaining frames are selected to highlight the elicited behavior. As a result, we observe that the deformation state of the tissue influences the overall dynamics differently. The first column highlights the variability in the AP wavelength, representing the spatial extension of the activation wave, which is due to the different repolarization states of the tissue induced by stress-assisted diffusion and stretch-activated currents. In particular, the AP wavelength varies as >6.2cm for case A, = 6.2cm for case B, and <2cm for cases C, D. In fact, when the *G*_*s*_ contribution is present, the excitation wave is much reduced with respect to the profiles generated with the electrophysiological three-variable model (1) and fine-tuned on experimental data. Such an effect is not present when *G*_*s*_ = 0.

Secondly, cases A and B (that is, where only *D*_1_ is activated) provide a similar behavior for spiral onset and case B shows the expected reduction in CV. Contrariwise, cases C and D (where also the contribution of *G*_*s*_ is present) induce much more complex dynamics, not expected in an isotropic medium. In particular, case C leads to a wave break and multiple spirals generation at the S2 stimulus that eventually collide and result in a single spiral wave. On the other hand, case D shows a more stable behavior generated by the presence of *D*_1_.

In addition, Table 3 also provides the minimum and maximum delay for the S2 stimulation (vulnerable window) allowing to induce a spiral wave in the uniaxially stretched tissue. It is evident that the presence of SAC reduces the minimum S2 stimulation time, $t_{{\text{S}}_{2}}^{\text{min}}$, by about 100*ms* with respect to the other cases and slightly increase the overall time span of the vulnerable window. Such a variation is motivated on the additional reaction current induced by the presence of Isac(λ, *u*) everywhere in the medium, but it is not expected from the experimental isochrones provided in Figure 1.

**Figure 8:**
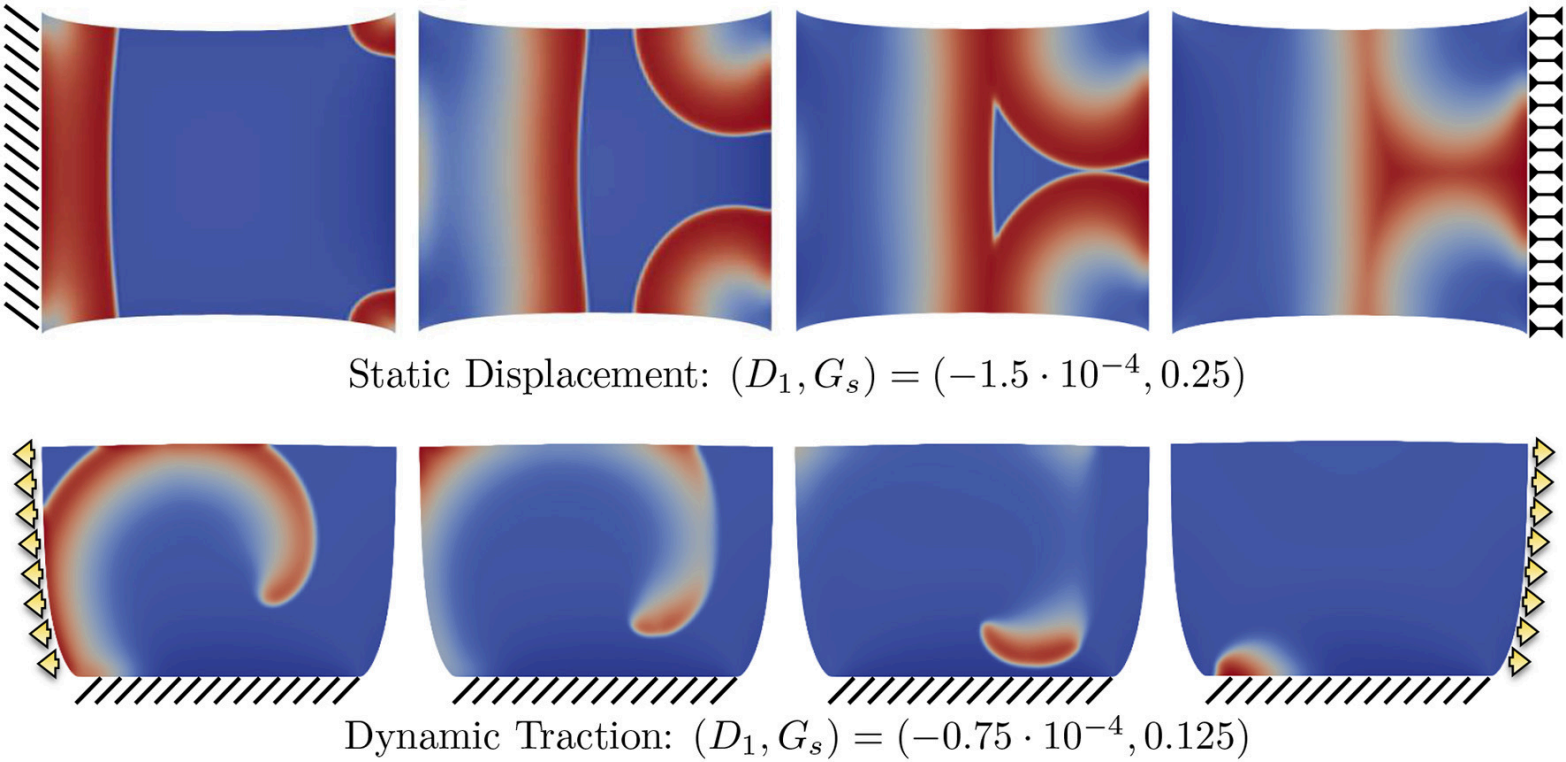
Example of different propagation patterns according to different mechanical boundary conditions and parameter space. First row shows the uniaxial static displacement configuration for which the selected parameters induce additional activations from the corners of the domain due to the excessive level of SAC (*G*_*s*_). Second row shows the dynamic traction configuration for which the initiated spiral wave goes through breakup due to the effect of mechanical loading.

To further corroborate this analysis, we provide in the top panels of Figure 8 an additional sequence referring to the combination $\left(D_{1},G_{s}\right)=\left(-1.5\cdot 10^{-4},0.25\right)$ in the case with static displacement boundary conditions, which falls in the range where no CV wave was measured. As anticipated, an excessive contribution due to SAC elicits extra activations where the stretch is maximum, i.e., at the corners of the domain. This particular behavior is not obtained when the stress-assisted contribution *D*_1_ is very high. Next, the bottom panels of Figure 8 show results using the combination $\left(D_{1},G_{s}\right)=\left(-0.75\cdot 10^{-4},0.125\right)$, which allows the quantification of CV but can eventually lead to spiral breakup and non-sustainability of the arrhythmic patterns due to the mechanical state of the tissue (corresponding to the case of dynamic traction, described below). This is a representative example of the key importance of boundary conditions and how MEF effects could be effectively translated into clinical studies.

### 3.3. Spiral drift and effects due to boundary conditions

**Figure 9:**
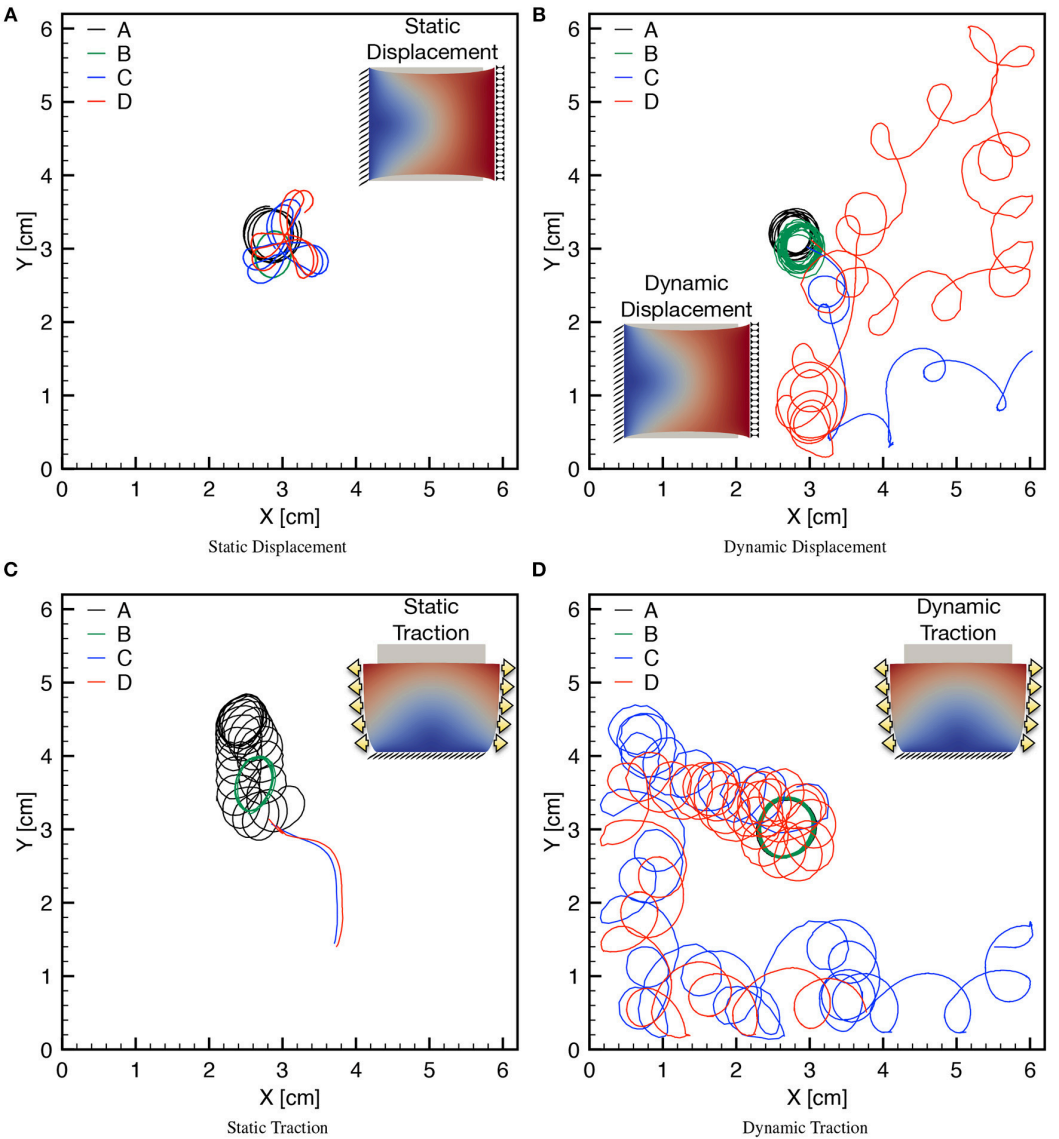
Tip trajectories for four combinations of MEF parameters (*D*_1_, *G*_*s*_) (see Table 3), applying static/dynamic–displacement/traction boundary conditions as indicated in the corresponding inset. Inset color code refers to the magnitude of the displacement field. **(A)** The last second of simulation is shown for the four cases with localized cores. **(B)** The last 3 s of simulations are shown highlighting the differences of the meandering. **(C)** Different times are shown for the four cases since for *G*_*s*_ > 0 the spirals exit the domain soon after initiation. **(D)** The last 3 s are shown for the case *G*_*s*_ > 0 highlighting the different meandering obtained with respect to *G*_*s*_ = 0. Minor discontinuities are due to the frame resolution for post processing analysis and are not linked to the accuracy of the numerical solution.

Finally, we turn to the analysis of meandering for the spiral tip for long run simulations (4s of physical time) comparing the four selected sets of parameters A,B,C,D in combination with static/dynamic–displacement/traction boundary conditions. In particular, we initiate the spiral wave via the S1-S2 stimulation protocol as discussed in the previous section, in absence of any mechanical loading such to start from the same initial conditions for each selected case. After spiral onset and stabilization (namely, for *t* > *t*_2_ = 250ms), we apply the following four different loadings:

- Static displacement: uniaxial displacement $\tilde{\varphi }={\left[0.1L,0\right]}^{T}$ applied on the right boundary while keeping the left one clamped (Figure 9A).
- Dynamic displacement: uniaxial time-dependent displacement $\tilde{\varphi }\left(t\right)={\left[0.1L\sin^{2}\left(\pi /400t\right),0\right]}^{T}$ applied on the right boundary while keeping the left one clamped (Figure 9B).
- Static traction: uniaxial sigmoidal time-dependent force ${\tilde{t}}_{i}\left(t\right)=t_{\max}\left[1.0-\exp\left(-\left(t-t_{2}\right)/5\right)\right]$ applied on the left and right boundaries while keeping the bottom side clamped (Figure 9C).
- Dynamic traction: uniaxial time-dependent force ${\tilde{t}}_{i}\left(t\right)=t_{\max}\sin^{2}\left(\pi /400t\right)$ applied on the left and right boundaries while keeping the bottom side clamped (Figure 9D).

For each mechanical loading, panels in Figure 9 show the trajectories of the spiral tip for the four MEF parameters combinations. Two important aspects are worthy of attention.

First, for each combination of the mechanical loading, the presence of the stress-assisted conductivity *D*_1_ tends to stabilize the meandering (see black and green traces). This behavior is particularly evident in Figure 9C where the combination $D_{1}=-0.75\cdot 10^{-4},G_{s}=0$ results into a localized core, while the case *D*_1_ = 0, *G*_*s*_ = 0 presents a circular, but slightly drifting core. Consequently, local stress-based heterogeneities appear in the medium when *D*_1_ is different from zero, leading to pinning-like phenomena also observed in Cherry and Fenton (2008), Cherubini et al. (2012), Jiménez and Steinbock (2012), and Liu et al. (2013). Moreover, these conditions are associated with an ellipsoidal shape of the core underlying the effective anisotropy induced by the stress-assisted coupling. All these observations agree with the conclusions from the extended analysis conducted on the chosen AP model in the original work from Fenton and Karma (1998b).

Secondly, when also SAC is present, the spiral meandering is unpredictable and strongly dependent on the applied boundary conditions (see blue and red traces). In this scenario, it is interesting to note that static loading induces a simple meandering which eventually pushes the spiral wave out from the domain (see Figure 9C), whereas dynamic conditions dictate a chaotic behavior that makes the spiral either to explore the whole domain, or to exit it. These patterns seem to be extreme conditions of hyper-excitability not expected in a two-dimensional isotropic medium (Fenton and Karma, 1998a; Fenton et al., 2002).

**Figure 10:**
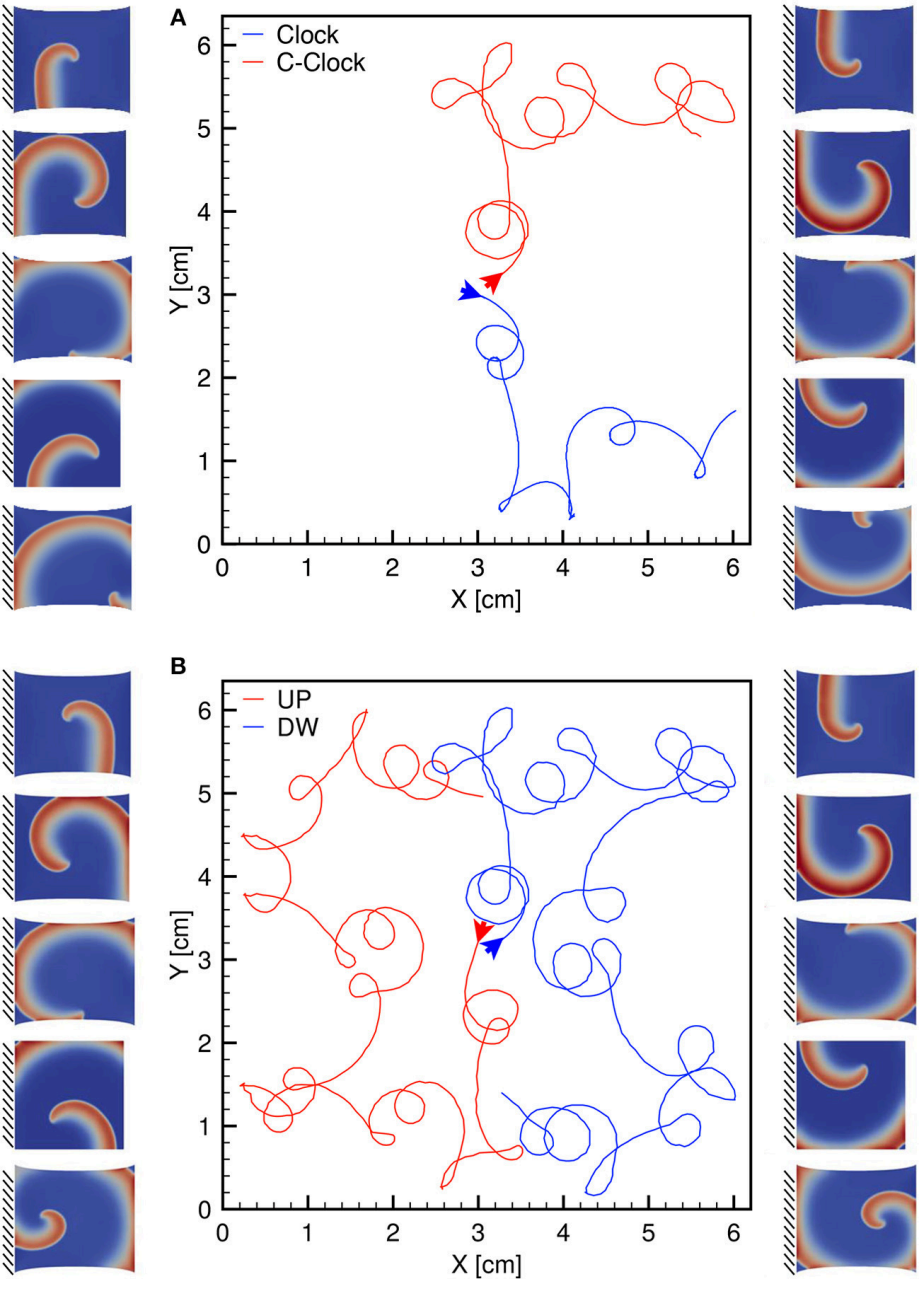
**(A)** Clockwise (blue) and counterclockwise (red) tip trajectories obtained in a dynamic uniaxially stretched case with MEF parameters *D*_1_ = 0, *G*_*s*_ = 0.125 and initiated via the S1-S2 stimulation protocol. **(B)** Counterclockwise spiral initiation from top (red) or bottom (blue) boundary. Side panels show progressive spiral frames for the two cases.

Finally, we highlight the symmetry of the observed behavior according to the clockwise or counterclockwise rotation of the spiral. This particular analysis is provided in Figure 10 and further links the excitation dynamics to the mechanical features. The different traces refer to the spiral core meandering observed for a dynamic uniaxially stretched case with MEF parameters *D*_1_ = 0, *G*_*s*_ = 0.125 and initiated via the S1-S2 stimulation protocol: case (a) compares a clockwise and counterclockwise spiral propagation; case (b) shows a counterclockwise spiral core initiated from the top (red) and bottom (blue) case. Corresponding sequences are also shown as side panels. This result is limited to the simplified nature of the domain adopted, i.e., 2D isotropic. A more realistic computational domain, embedding fiber directionality and tissue thickness, would show more involved dynamics in a complex spatiotemporal and clinical relevant perspective.

## 4. Conclusion

We have advanced a minimal model for the electromechanics of cardiac tissue, where the mechano-electrical feedback is incorporated through two competing mechanisms: the stretch-activated currents commonly found in the literature, and the stress-assisted diffusion (or stress-assisted conductivity) recently proposed by Cherubini et al. (2017). Both the electrophysiology and the mechanical response adopt a phenomenological simplified description, but a preliminary validation is provided through a set of numerical simulations that agree qualitatively with a set of experimental data for pig right ventricle.

The implications of the intensity and degree of nonlinearity assumed for the stress-assisted diffusion effect are studied from the viewpoint of changes in the conduction velocity and the dynamics of spiral waves in simplified 2D domains. Multiple electrical stimulations protocols and non-trivial mechanical loadings have been investigated highlighting the strong coupling due to the different MEF contributions. The analysis supports the hypothesis that the simplistic formulation adopted for stretch-activated currents seems to deviate from the experimental evidence, in line with recent contributions addressing the coupled modeling of SACs and stretch-induced myofilament calcium release at the myocyte level (Timmermann et al., 2017). On the other hand, in a homogenized setting, the stress-assisted diffusion formulation produces a series of interesting phenomena that qualitatively match heterogeneities and anisotropies observed during mechanical stretching of pig right ventricle via fluorescence optical mapping.

Limitations of the present work are partially linked to the phenomenological approach adopted to describe the complex multiscale mechanisms intrinsic in the cardiac tissue and partially due to the simplified computational domain. In this regards, we aim at investigating more reliable stretch-activated current formulations leading to alternans behaviors (Galice et al., 2016) within a multiscale mechanobiology perspective (Nava et al., 2016; Stålhand et al., 2016; Cyron and Humphrey, 2017) and tacking into account the intracellular calcium cycling influenced by mechanical stretch, because all these effects have been proposed as concurring mechanisms of arrhythmogenesis within the heart. From the mechanical point of view, we mention as main limitation the adoption of a simplified isotropic hyperelastic material model which can be generalized to more complex and reliable formulations. This will include, for example, active strain anisotropies, muscular and collagen fiber distributions in an orthotropic mechanical framework that the authors have been extensively developing during the last decade (Cherubini et al., 2008; Nobile et al., 2012; Gizzi et al., 2015, 2016, 2018; Pandolfi et al., 2016). Such a generalization will maintain the nature of the present theoretical framework in terms of MEF competing effects. In this line, we also aim to generalize our theoretical and computational approach toward intrinsic multiscale and multiphysics mechano-transduction problems (Weinberg et al., 2017; Lenarda et al., 2018), e.g., the uterine smooth muscle activity (Young, 2016; Yochum et al., 2017) or the intestine biomechanics activity (Pandolfi et al., 2017; Brandstaeter et al., 2018) by implying the usage of network approaches (Giuliani et al., 2014; Robson et al., 2018) and data assimilation procedures (Barone et al., 2017). In addition, the investigation of the complex spatiotemporal dynamics, chaos control and multiphysics couplings in excitable systems (see e.g., Hörning et al., 2017; Christoph et al., 2018) can be emphasized within the proposed electromechanical framework by using realistic three-dimensional cardiac structures (Lafortune et al., 2012). We also mention implications of the proposed models in the mathematical study of general stress-assisted diffusion problems, as recently carried out in Gatica et al. (2018). Finally, we hope that the present contribution may open new experimental studies to translate the complex MEF phenomena into the clinical practice (Meijborg et al., 2017; Orini et al., 2017) identifying novel risk indices for cardiac arrhythmias (Gizzi et al., 2017).

## Ethics statement

All experiments conform to the current Guide for Care and Use of Laboratory Animals published by the National Institutes of Health (NIH Publication No. 85–23, revised 1996), and approved by the Office of Research and Integrity Assurance at Georgia Tech.

## Author contributions

AL, AG, RR-B, CC, FF, and SF design of the study; AL, AG, and RR-B numerical methods and computational tests; FF experimental measurements; AL and AG Statistical analysis; AL, AG, RR-B, CC, FF, and SF Manuscript writing.

### Conflict of interest statement

The authors declare that the research was conducted in the absence of any commercial or financial relationships that could be construed as a potential conflict of interest.
